# Monitoring sedation for bronchoscopy in mechanically ventilated patients by using the Ramsay sedation scale versus auditory-evoked potentials

**DOI:** 10.1186/1471-2466-14-15

**Published:** 2014-02-06

**Authors:** Chien-Wei Hsu, Shu-Fen Sun, Kuo-An Chu, David Lin Lee, Kam-Fai Wong

**Affiliations:** 1 Intensive Care Unit, Department of Medicine, Kaohsiung Veterans General Hospital, 386 Ta-Chung First Road, Kaohsiung City 813, Taiwan; 2 Medicine Department, School of Medicine, National Yang-Ming University, 155 sec.2 Linong Street, Taipei City 112, Taiwan; 3 Department of Physical Medicine and Rehabilitation, Kaohsiung Veterans General Hospital, 386 Ta-Chung First Road, Kaohsiung City 813, Taiwan; 4 Chest Medicine, Department of Medicine, Kaohsiung Veterans General Hospital, 386 Ta-Chung 1st Road, 813 Kaohsiung City, Taiwan; 5 Institute of Statistics, National University of Kaohsiung, 700 Kaohsiung University Road, Nanzih District, Kaohsiung City 811, Taiwan

**Keywords:** Auditory-evoked potential, Bronchoscopy, Critical care, Ramsay sedation score, Sedation

## Abstract

**Background:**

Appropriate sedation benefits patients by reducing the stress response, but it requires an appropriate method of assessment to adjust the dosage of sedatives. The aim of this study was to compare the difference in the sedation of mechanically ventilated patients undergoing flexible bronchoscopy (FB) monitored by auditory-evoked potentials (AEPs) or the Ramsay sedation scale (RSS).

**Methods:**

In a prospective, randomized, controlled study, all patients who underwent FB with propofol sedation were monitored and their sedation adjusted. During FB, one group was monitored by AEP and another group was monitored by RSS. The propofol dosage was adjusted by the nursing staff during examination to maintain the Alaris AEP index (AAI) value between 25 and 40 in the AEP group and the RSS at 5 or 6 in the RSS group. Before FB and during FB, the AAI, heart rate (HR), and mean arterial pressure (MAP) were recorded every 5 min. The percentages of time at the sedation target and the propofol dosages were calculated.

**Results:**

Nineteen patients received AEP monitoring and 18 patients received RSS monitoring. The percentage of time at the sedation target during FB was significantly higher in the AEP monitoring group (51.3%; interquartile range [IQR], 47.0–63.5%) than in the RSS group (15.4%; IQR, 9.5–23.4%), (*P* < 0.001). During FB, the RSS group had a significantly higher AAI (*P* = 0.011), HR (*P* < 0.001), and MAP (*P* < 0.001) than the AEP group.

**Conclusions:**

In mechanically ventilated patients undergoing FB, AEP monitoring resulted in less variation in AAI, HR, and MAP, and a higher percentage of time at the sedation target than RSS monitoring.

**Trial registration:**

ClinicalTrials.gov NCT01448811.

## Background

A patient who undergoes bronchoscopy frequently suffers from pain, cough, and dyspnea, and may remember the procedure as an unpleasant experience [1,2]. Sedation is suggested for patients undergoing flexible bronchoscopy (FB), unless contraindications exist [3]. Sedation benefits patients by reducing the stress response, thereby improving a patient’s tolerance of medical procedures [4]. Appropriate sedation requires a good method of assessment to adjust the dosage of sedatives. However, there is no consensus regarding the best tool to evaluate sedation or how frequently sedation should be used [5,6]. In the intensive care unit (ICU), the Ramsay sedation scale (RSS) is a traditional method used to assess the sedation level [7]. Middle latency auditory-evoked potentials (MLAEPs) measure the output of the central nervous system in response to auditory signals, and appear to be a method for estimating the depth of sedation [8]. Middle latency auditory-evoked potentials reflect changes in electroencephalogram waves and represent the earliest cortical response to acoustic stimuli [9]. The measurement of MLAEPs can be used to monitor continuously the consciousness level by auditory stimuli to the brain and can be measured quantitatively by using the Alaris auditory-evoked potential index (AAI). An AAI level above 60 indicates that a patient is fully awake; a level between 40 and 60 indicates light to moderate sedation, a level between 25 and 40 indicates deep sedation; and a level between 15 and 25 is satisfactory for surgery [10]. Most anesthetics depress MLAEPs in a dose-dependent fashion [11], and the changes are independent of the presence of opioids [12]. In this study, we aimed to compare the difference of sedation in mechanically ventilated patients undergoing FB who were monitored by AEP or by RSS.

## Methods

### Study design

Between March 2007 and March 2008, a prospective, randomized, controlled trial was conducted at the adult ICU of a tertiary medical center with 77 adult ICU beds. The Institutional Review Board of the Kaohsiung Veterans General Hospital (Kaohsiung City, Taiwan) approved the trial and consent forms. The patients or their next of kin provided informed consent. Procedures were performed in accordance with the Helsinki Declaration.

### Subjects

**Figure 1:**
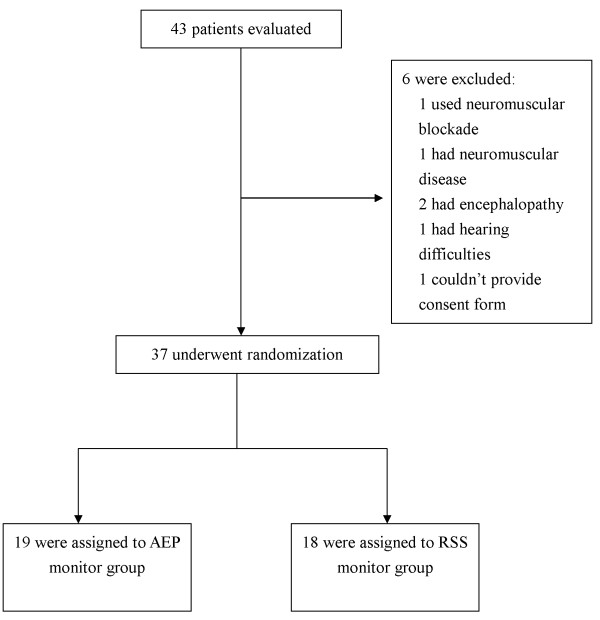
**Assessment and randomization of the study patients.** See Table 1 for detailed characteristics of the randomized patients.

Mechanically ventilated patients in the ICU, aged 18 or over, who needed a FB and did not have contraindications for FB were eligible for this study. Patients needed a FB if they had abnormal chest radiography findings such as a mass, nodule, or collapse, inflammation in the lung that needed evaluation of a possible lung infection; blood in the sputum; or foreign body in the airway. Exclusion criteria included patients with pacemakers, neuromuscular blockade, neuromuscular diseases with motor dysfunction, neurological disease, encephalopathy, hypothermia, hyperthermia, propofol allergy, or hearing difficulties. After applying the inclusion and exclusion criteria, 37 patients were randomized to the AEP group or the RSS group by software that generated a random number without blocking (Figure 1). Except for the interventionists, the patients and other staff members (e.g., doctors and assistants) were not informed of the group assignment.

### Intervention

Data included the reason for the FB, patient’s age, body weight, gender, and acute physiology and chronic health evaluation II score [13] were recorded before intervention. Analgesia was provided by a continuous infusion of fentanyl. The dosage was adjusted to reach adequate analgesia, based on a visual analog scale. Fentanyl dosages were recorded. Propofol was administered by an infusion pump (XLD, Abbott, Abbott Park, USA). No other sedative or analgesic medication was administered.

### Phase 1: Preparation for flexible bronchoscopy

**Figure 2:**
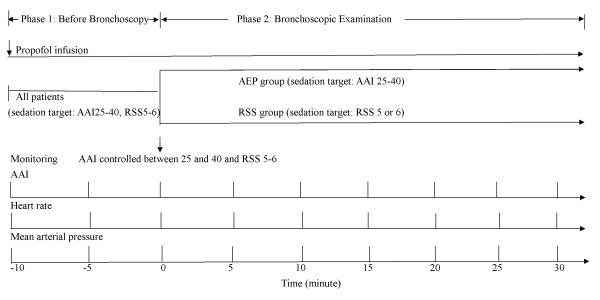
**Design of the procedure.** In all patients, the target sedation level before bronchoscopy was an Alaris auditory-evoked potential index (AAI) level between 25 and 40 and a Ramsay sedation scale (RSS) of 5 or 6. During bronchoscopy, patients were randomized to the auditory-evoked potentials (AEP) group or the RSS group. The sedative was adjusted in accordance with the AAI level or the RSS level. The AAI, heart rate, and mean arterial pressure were recorded every 5 min before flexible bronchoscopy and after flexible bronchoscopy.

Each patient had an indwelling arterial line, and the MAP was measured. The heart rate (HR) was determined by continuous electrocardiography. All patients were ventilated using the assisted-controlled mode and were monitored with pulse oximetry. One hundred percent inspired oxygen was supplied to maintain an arterial oxygen saturation greater than 90% during the intervention. Before the FB, all patients were connected to the AEP monitor (Alaris Medical Systems, Danmeter A/S, Odense, Denmark). The electrodes were positioned at the mid-forehead, the left forehead, and the left mastoid after the skin was cleaned with alcohol. The target of sedation adopted deep sedation because most FB procedures were advanced diagnostic or therapeutic bronchoscopy procedures. All procedures were administered to mechanically ventilated patients in the ICU. Transbronchial brushing, biopsy, or lavages were administered to patients with pneumonia or lung tumor. Foreign removal was administered to one patient with foreign body aspiration. The target of AAI level was between 25 and 40 and the target RSS value [14] was 5 or 6. These targets were chosen because the electroencephalogram sedation scale range of 40-25 corresponds to the RSS range of 5 to 6 [15]. Before the FB procedure, the AAI, HR, and MAP were recorded every 5 minutes. The propofol dosages were adjusted to maintain the AAI level between 25 and 40 and the RSS at 5 or 6. Electromyographic (EMG) activity was also monitored. Figure 2 shows the design of the procedure.

### Phase 2: Flexible bronchoscopy examination

An experienced respiratory physician performed the FB. The distal end of the endotracheal tube was connected to an adaptor that allowed the maintenance of mechanical ventilation during the procedure. Flexible bronchoscopy began when the AAI level was controlled between 25 and 40 and the RSS was at 5 or 6. The bronchoscope was passed into the trachea through the adaptor and endotracheal tube. Topical lidocaine 2% was used by the spray-as-you-go technique on the bronchial mucosa during the FB examination. The AAI, HR, and MAP were recorded every 5 minutes during bronchoscopy. In the AEP monitoring group, trained ICU nurses adjusted the propofol dosage based on the AAI levels. If AAI was greater than 40, propofol was increased; if the AAI was less than 25, the dosage was decreased. In the RSS monitoring group, trained ICU nurses adjusted the propofol dosage based on the RSS level. The RSS was controlled at 5 or 6. Alaris AEP index monitoring was also administered to the RSS monitoring group. However, the AEP monitor was shielded and the trained ICU nurses who adjusted the propofol dosage were unaware of the AAI levels. The AAI levels were censored and recorded if the RSS, HR and MAP were checked. Each adjustment increased or decreased 10%–20% infusion doses of propofol [16]. The mean propofol dosage before and after the examination, the times of propofol dosage adjustment, and the interval from the beginning of the FB to the first propofol dosage adjustment were recorded.

### Outcomes

The primary endpoint of this study was to compare the differences in the AAI between the AAI monitoring group and the RSS monitoring group. The secondary endpoint was to compare the differences between the AAI monitoring group and the RSS monitoring group in the HR; MAP; times of propofol dosages adjustment; time to the first propofol dosages adjustment; mean propofol dosage during the examination; percentage of change in the propofol dosage from the baseline dose; percentage of time at the sedation target; percentage of AAI level greater than 40; and occurrence of significant hypotension.

The percentage of time at the sedation target is defined as the percentage of minutes in which a patient maintains an adequate or desired level of sedation, based on the assessment method used [16]. The equation is as follows: the percentage of time at the sedation target (%) = (adequate sedation minutes/total minutes of sedation) × 100.

### Statistical analysis

We performed a power calculation to determine the ideal sample size. A minimum of 18 patients was required in each group to detect a difference in the HR with a power of 90% and a confidence interval of 95%. This was based on a previous study involving HR and different sedation levels during FB [17].

All data were analyzed by SPSS version 12.0 (SPSS, Inc., Chicago, IL). The data were presented as the mean ± standard deviation (SD), the median [interquartile range], or the number and percentages.

The Mann–Whitney *U* test was used to compare continuous variables. The chi-square test or Fisher’s exact test was used to compare dichotomous variables, depending on the expected frequency of occurrence. The correlation between the AAI and the RSS, HR, and MAP were analyzed by Spearman correlation analysis. Changes in the AAI, HR, and MAP were analyzed with a generalized linear model for repeated measures by using dummy variables. A *P* < 0.05 was considered statistically significant.

## Results

### Patient characteristics

Of the 37 patients included in the study, 19 received AEP monitoring and 18 received RSS monitoring (Figure 1). Table 1 shows the baseline characteristics of all patients before FB. There were no significant differences between the two groups.

**Table 1:** Demographic data of all patients

**Characteristics**	**AEP monitor group (*n*** **= 19)**	**RSS monitor group (*n*** **= 18)**	**P**
Admission diagnosis			
Pneumonia	10	11	
Sepsis	3	2	
Malignancy	2	2	
Stroke	2	0	
Burn	0	1	
Others	2	2	
Reasons for bronchoscopy			
Pneumonia	13	12	
Lung cancer	4	3	
Hemoptysis	1	1	
Sputum impaction	1	0	
Foreign body aspiration	0	1	
Burn	0	1	
Age (yr)	68.6 ± 14.1	68.8 ± 16.2	0.968
Body weight (kg)	63.4 ± 8.4	58.5 ± 11.2	0.148
Gender (F/M) (%)	5/14 (26.3)	4/14 (28.6)	0.759
APACHE II score	23 [20-27]	22 [20-25]	0.700
ICU day when bronchoscopy was performed	5 [2.5-9.5]	4.5 [2-7]	0.399
PaO_2_/FiO_2_ before bronchoscopy	204.9 ± 32.8	212.9 ± 32.9	0.464
Heart rate (beats/min)	96 ± 24	94 ± 17	0.728
MAP (mmHg)	87 ± 18	88 ± 11	0.859
AAI	55 [49-60]	53 [46-60]	0.382
RSS	3 [2.5-4]	3 [2.25-4]	0.930
Propofol dosage before bronchoscopy (μg⋅kg^-1^⋅min^-1^)	16.8 [11.4-32.5]	17.6 [10.2-21.2]	0.617

Data are presented as the number (*n)*, mean ± standard deviation, or median [interquartile range]. *AAI*: Alaris AEP index; *AEP*: auditory evoked potentials; *APACHE*: acute physiologic and chronic health evaluation; *FiO*_*2*_: fraction of inspired oxygen; *MAP*: mean arterial pressure; *PaO*_*2*_: partial pressure of arterial oxygen; *RSS*: Ramsay sedation scale.

### Differences in the propofol dosage during FB

Table 2 shows the differences between the 2 groups during FB. The AEP group had a significantly earlier and greater number of propofol dosage adjustments, compared with the RSS group. The median propofol dosage during examination was higher in the AEP group than in the RSS group. The median propofol dosages increased more in the AEP group than in the RSS group (Table 2). The percentage of time at the sedation target during FB was higher in the AEP group than in the RSS group. The percentage of AAI levels greater than 40 was higher in the RSS group than in the AEP group. During FB, there was no significant difference between the 2 groups in the fentanyl dosages, duration of FB, or number of patients with significant hypotension and EMG activity. However, there was a trend toward greater hypotension in the AEP group.

**Table 2:** Differences between two groups during bronchoscopic examination

**Variables**	**AEP monitor group (*n*** **= 19)**	**RSS monitor group (*n*** **= 18)**	**P**
Median number of propofol dosage adjustment	6 [5-7]	3 [2-3.8]	<0.001
Time to the first adjustment of the propofol dosage (second)	137 [117.5-200.5]	466 [376.5-553.5]	<0.001
Propofol dosage during examination (μg⋅kg^-1^⋅min^-1^)	43.5 [28.5-49.3]	24.2 [15.5-32.4]	0.025
Fentanyl dosage during examination (μg⋅kg^-1^⋅hr^-1^)	2.5 [1.8-3.1]	2.7 [1.9-3.4]	0.679
Percentage of propofol dosage change compared with the baseline dose (%)	93.5 [48.4-172.3]	39.5 [30.4-53.9]	0.011
Percentage of time at sedation target (%)	51.3 [47.0-63.5]	15.4 [9.5-23.5]	<0.001
Percentage of AAI levels greater than 40 during examination (%)	35.3 [27.1-51.5]	84.5 [76.5-90.4]	0.033
Patients with significant hypotension (MAP less than 60 mmHg) (%)	2 (10.5)	0 (0)	0.154
Duration of FB examination (min)	29 [27-32]	30 [28-32]	0.860
EMG (dB)	18 [16-23.5]	20 [17-25]	0.751

Data are presented as the number (*n)*, or median [interquartile range]. *AAI*: Alaris AEP index; *AEP*: auditory evoked potentials; *dB*: decibel; *EMG*: electromyography; *FB*: flexible bronchoscope; *MAP*: mean arterial pressure *RSS*: Ramsay Sedation Scale.

### Between group differences in the AAI, HR, and MAP

**Figure 3:**
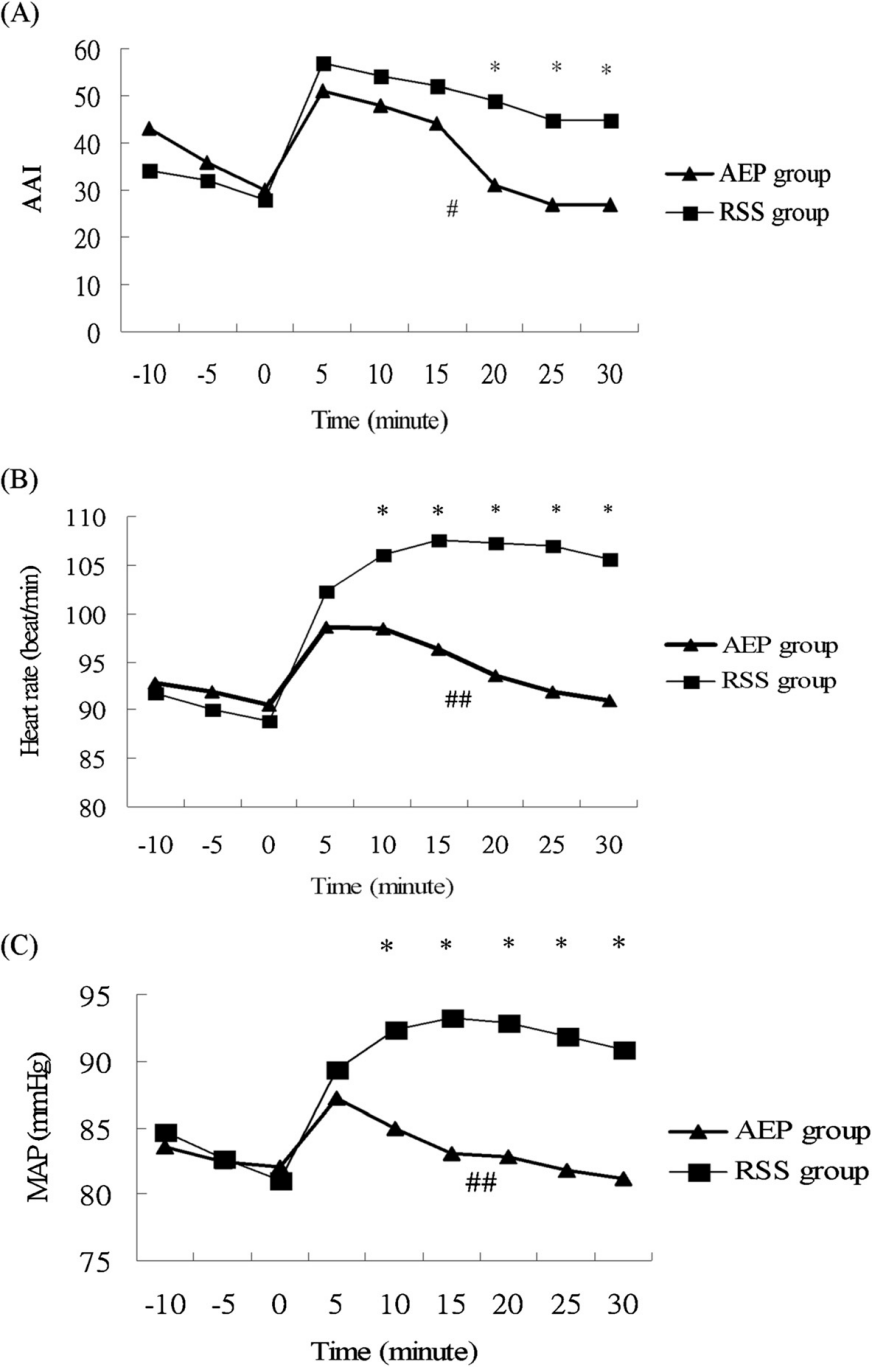
**Differences between the auditory-evoked potentials (AEP) monitoring group and Ramsay sedation scale (RSS) monitoring group in (A) the Alaris auditory-evoked potentials index (AAI), (B) the heart rate, and (C) the mean arterial pressure (MAP).** A generalized linear model of repeated measures shows a statistical significance between the groups. * *P* < 0.05 for two groups at different time. # *P* = 0.011 and ## *P* <0.001 for the entire flexible bronchoscopic examination period.

In both groups, the AAI, HR, and MAP increased within 5 minutes after FB began (Figure 3A-C). After the examination began in the AEP group, the AAI, HR, and MAP returned to their baseline values in 20 min, 20 min, and 10 min, respectively after the examination began (Figure 3A-C). In the RSS group, the AAI, HR, and MAP did not return to their baseline values, and they remained higher than their baseline values during the whole examination period.

Using dummy variables, a generalized linear model for repeated measures revealed a significantly higher AAI (*P* = 0.011), HR (*P* <0.001) and MAP (*P* < 0.001) in the RSS group than in the AEP group during the course of the FB examination. Significant differences in the AAI, HR, and MAP between the two groups were present after 20 min, 10 min, 10 min, respectively, after the examination began (*P* < 0.05) (Figure 3A-C).

### Correlation between the AAI and the RSS, HR, and MAP

**Figure 4:**
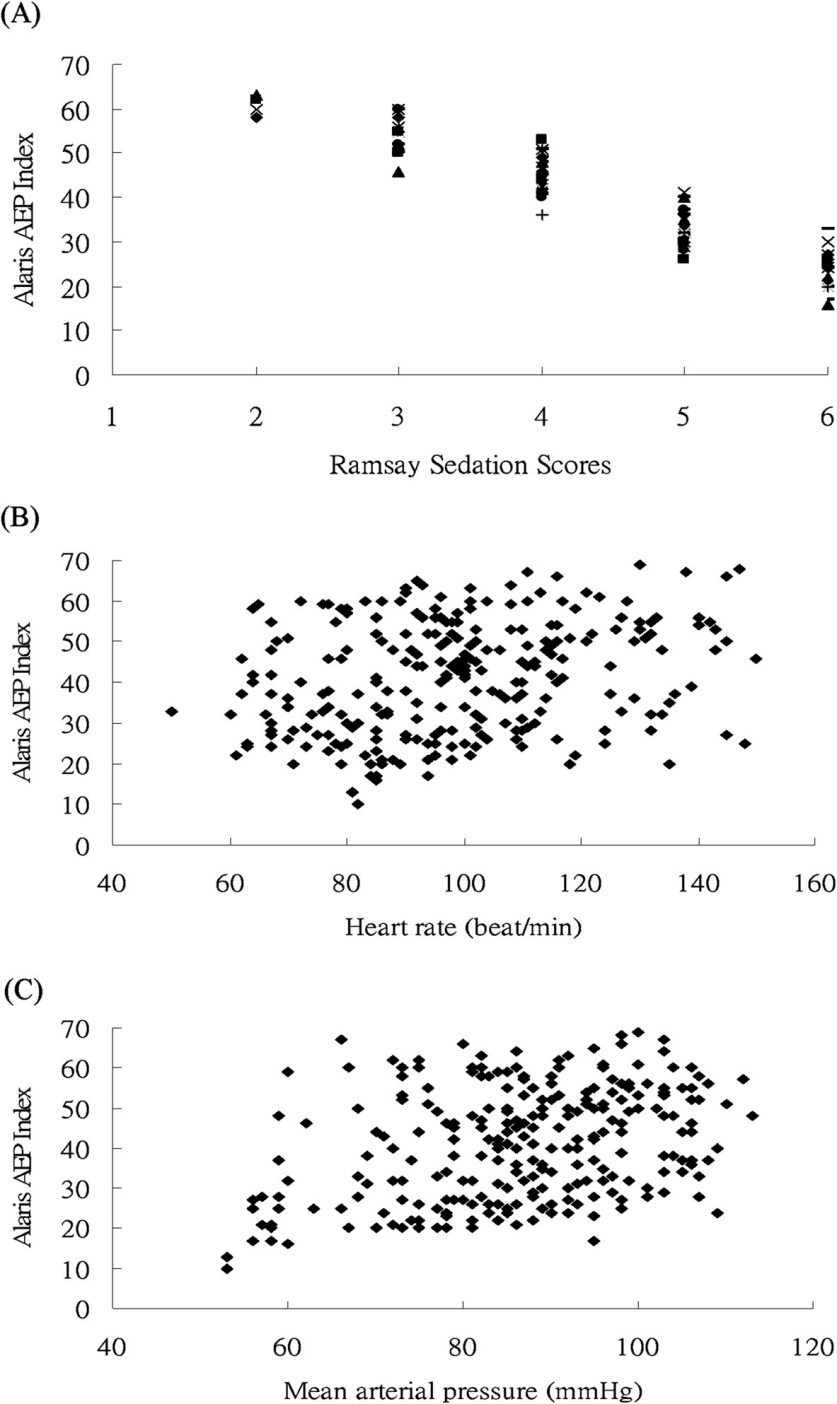
**The values of the (A) the Ramsay sedation scores, (B) the heart rate, and (C) the mean arterial pressure and the corresponding Alaris auditory-evoked potentials index (AAI) value.** The AAI is correlated with the Ramsay sedation scores, heart rate, and mean arterial pressure (MAP) (for all, *P* < 0.001). Spearman’s *rho* coefficients between the AAI and the RSS, HR, and MAP were -0.949, 0.255, and 0.337, respectively.

Figure 4A-C demonstrate the concomitant AAI values when RSS, HR and MAP were measured. There were significantly negative and positive correlations between the AAI and the RSS, HR, and MAP (all *P* < 0.001). The Spearman correlation coefficients between the AAI and the RSS, HR, and MAP were -0.949, 0.255, and 0.337, respectively. The RSS had the best correlation with the AAI.

## Discussion

This study showed that, when FB was administered to mechanically ventilated patients, the patients who underwent AEP monitoring had a significantly higher percentage of time at the sedation target, compared with patients who underwent RSS monitoring. During the course of the FB examination, patients monitored with AEP used higher sedative dosages and had less change in the AAI, compared with patients monitored with RSS. Patients monitored with RSS had a higher percentage of AAI levels greater than 40, indicating that most of these patients were inadequately sedated and the goal of deep sedation was not reached most of the time.

Undersedation can result in tachycardia and hypertension, which can lead to adverse outcomes in ICU patients [18,19]. The reasons for undersedation with RSS monitoring may be the following: (1) RSS is an intermittent monitoring procedure and requires more time to achieve the sedation goal because of the nature of discontinuous monitoring; FB is a short-term examination, and it is often finished before patients reached the sedation goal, thus resulting in the significantly lower percentage of time at the sedation target in the RSS group; (2) AEP and RSS require a stimulus; AEP is automated but RSS requires human intervention with the potential for variation in intensity; (3) the lag time from the stimulus to the response may be longer for RSS monitoring since it is an observational assessment that examines the patient’s responsiveness to stimuli; RSS requires a practitioner to be at the bedside with some time to do the sedation assessment.

Flexible bronchoscopy is an important tool for the diagnosis of pulmonary disease, especially infectious pneumonia [20]. However, it is an uncomfortable examination, resulting in a significant rise in the HR and blood pressure [17]. We found that the AAI, HR, and MAP increased quickly once the bronchoscope was inserted into the endotracheal tube. These parameters could recover if the deep sedation goal were attained. Medical procedures for ICU patients increase metabolic demand and increase the output of the cardiovascular system. Sedatives suppress the metabolic and hemodynamic response, and they reduce oxygen consumption and autonomic hyperactivity [4,21].

Some studies have shown that AEP is correlated well with the RSS in nonparalyzed patients [15,22,23]. Our study had similar findings. The HR and MAP were also correlated with AEP. The AEP had a better correlation with the RSS than with the HR or MAP. The HR and blood pressure are not specific or sensitive markers of the sedation level in critically ill patients [5]. Changes in the HR and blood pressure are attributable to many factors, other than sedation [9].

The advantages of the RSS are that it can be performed at the bedside and it is easily reproducible [24,25]. However, RSS is a subjective evaluation. It has attracted criticism because of the lack of clear discrimination and specific descriptors to differentiate between the various levels [26,27], because of the problem of inter-rater variation in interpretation [28], and because its usefulness is limited in patients receiving neuromuscular blockades [22].

Auditory-evoked potential monitoring has the advantages of continuous monitoring without inter-rater variation in interpretation, and it can be used in patients receiving neuromuscular blockades [24]. Auditory-evoked potentials provide a clear assessment of the depth of sedation, although AEP is influenced by muscle activity, which increases MLAEP values [29]. In addition, auditory stimuli over long periods likely disturb patients, especially patients under light sedation. This indicates that AEP should be monitored intermittently when prolonged monitoring is required [30].

The ideal level of sedation varies for different situations, and the adjustment of dosage should always be considered when a patient’s needs change [4,18]. Frequent evaluation and adjustment is an integral component of most patient-focused management algorithms [31]. Therefore, continuous monitoring of sedation is important for patients with critical illnesses; AEP monitoring allows this. Optimizing sedation can protect patients from wide variations in blood pressure, agitation, and secondary organ injury [9].

Several limitations exist in this study. First, patients with neuromuscular blockades were not included. The RSS is a numerical scale of motor responsiveness that is graduated in accordance with increasing depth of sedation. Therefore, it cannot evaluate the level of sedation accurately in patients with neuromuscular blockade. In this study, we therefore excluded paralyzed patients to avoid inaccuracy. Second, patient movement can cause EMG artifacts and affect the AAI levels. To avoid EMG artifacts, as much as possible we did not move patients or administer to them a clinical stimulus during the study period. Third, all patients were supported by mechanical ventilation. Thus, the results may not be generalized to patients without mechanical ventilation. Furthermore, we studied patients receiving FB in this study. Different procedures in the ICU may have different characteristics and need a different assessment procedure. Further studies are needed for other invasive procedures to determine the appropriate sedative assessment tool in the ICU patients.

## Conclusions

Compared with RSS monitoring, AEP monitoring provided better sedation monitoring and allowed a more appropriate sedative adjustment to reach the sedative goal in mechanically ventilated patients undergoing FB. Patients monitored with AEP have a significantly higher percentage of time at the sedation target and less variation in the AAI, HR, and MAP, compared with patients monitored with RSS.

## Abbreviations

AAI: Alaris auditory-evoked potential index; AEP: Auditory-evoked potentials; EMG: Electromyography; FB: Flexible bronchoscopy; HR: Heart rate; ICU: Intensive care unit; MAP: Mean arterial pressure; MLAEP: Middle latency auditory-evoked potential; RSS: Ramsay sedation scale.

## Competing interests

The authors have no competing interests to declare.

## Authors’ contributions

C-WH was the main contributor to the study design, data interpretation, and manuscript drafting. S-FS contributed to data acquisition and analysis and manuscript revision. K-AC and D-LL contributed to the execution of the study, and K-FW contributed to the statistical analysis of data. All authors read and approved the final manuscript.

## Pre-publication history

The pre-publication history for this paper can be accessed here:

http://www.biomedcentral.com/1471-2466/14/15/prepub
